# Live Imaging of Phosphate Levels in Arabidopsis Root Cells Expressing a FRET-Based Phosphate Sensor

**DOI:** 10.3390/plants9101310

**Published:** 2020-10-03

**Authors:** Ana G. L. Assunção, Sisse K. Gjetting, Michael Hansen, Anja T. Fuglsang, Alexander Schulz

**Affiliations:** 1 Department of Plant and Environmental Sciences, Copenhagen Plant Science Center, University of Copenhagen, 1871 Frederiksberg C, Denmark; sg@plen.ku.dk (S.K.G.); mh@plen.ku.dk (M.H.); atf@plen.ku.dk (A.T.F.); als@plen.ku.dk (A.S.); 2 CIBIO-InBIO—Research Center in Biodiversity and Genetic Resources, University of Porto, 4485-661 Vairão, Portugal

**Keywords:** *Arabidopsis thaliana*, phosphate, FRET sensor, FLIPPi, Pi homeostasis, plant nutrition

## Abstract

Phosphorous (P) is an essential macronutrient in all organisms serving various fundamental biological processes, and is one of the least available plant nutrients in the soil. The application of inorganic phosphate (Pi) fertilizers is frequent, but it has a high environmental and financial cost. Breeding crops for improved Pi use-efficiency is a promising plant-based solution to pursue a reduction of fertilizer dependency. Availability of tools for monitoring changes of plant cellular Pi concentration in real-time can contribute to advancing knowledge on the molecular basis of Pi transport and homeostasis in plants. Genetically encoded fluorescent sensors have provided new insight on cellular processes. Here, we show that two Pi Fluorescence Resonance Energy Transfer (FRET)-based sensors from the FLIPPi family, the low-affinity FLIPPi-30m and the high-affinity FLIPPi-4µ, can be expressed and analyzed in *Arabidopsis thaliana* with wild-type background. These FLIPPi sensors had not been tested in plants, but only in mammalian cell lines. We show FRET response and live imaging of Pi levels in seedling roots of Arabidopsis FLIPPi-30m and FLIPPi-4µ lines. Our results reinforce that sensors from the FLIPPi family are valuable tools for studying mechanisms of Pi transport and homeostasis in plants, and for research towards a more sustainable use of Pi fertilization.

## 1. Introduction

Phosphorous (P) is an essential macronutrient in all organisms, being required at low millimolar range in living cells. It is a component of nucleic acids, phospholipids and ATP, and plays key roles in signal transduction cascades and regulation of enzyme activities [1,2,3]. Plants acquire P in its ionic, oxidized form, as inorganic phosphate (orthophosphate; Pi), and although P is relatively abundant in ecosystems, it is one of the least available plant nutrients in the soil. Precipitation and mineralization processes render it relatively immobile and not readily available to roots, with Pi concentrations in soil solution often being at low micromolar ranges (2–10 µM) [1,4]. To overcome the limited supply of Pi, plants have evolved adaptive mechanisms to increase Pi availability, uptake and use-efficiency. Such mechanisms include root morphological and architectural changes, enzyme-mediated Pi mobilization, transcriptional activation of high-affinity Pi transporters and symbiotic relationship with mycorrhizal fungi [3,5,6,7]. In agriculture, crops receive frequent Pi fertilization in order to maintain yield and quality, but this has a high environmental and financial cost. Runoff of excess fertilizer into surface waters has a negative environmental impact. In addition, the production of Pi fertilizer is unsustainable due to high energy costs and non-renewable, finite reserves of phosphate rock [8]. Breeding crops that acquire and use Pi more efficiently is a promising plant-based solution to reduce fertilizer dependency. Availability of tools to monitor the dynamics and changes in cellular Pi concentrations can contribute to study transport mechanisms and unravel regulatory networks of Pi homeostasis and adaptation to low-Pi stress [3,9].

Genetically encoded fluorescent sensors, or biosensors, are powerful tools that have provided new insight on cellular processes [10]. These biosensors monitor their targets (including ions, metabolites, or enzymatic activity) with high spatial and temporal resolution, and with cellular and subcellular localization [11]. One class of biosensors are the Fluorescence Resonance Energy Transfer (FRET) sensors that rely on the FRET status change between two fluorescent molecules (most commonly variants of Green Fluorescent Protein, GFP) that are covalently linked to the sensing domain. Upon binding of a target molecule (ligand) to the sensing domain, the sensor undergoes a conformational change causing a change in FRET efficiency that can be quantified [12]. A variety of FRET-based sensors have been generated [13], mainly in mammalian systems [14], but with examples in plants in the analysis of metabolites such as glucose, sucrose [15,16,17], amino acid glutamine [18], or pH [19].

The Pi FRET-based sensors, named Fluorescent Indicator Protein for inorganic Phosphate (FLIPPi), are genetically encoded fluorescent sensors that consists of a Pi binding bacterial periplasmic Binding Protein (PiBP), derived from cyanobacteria *Synechococcus sp.*, fused to an enhanced Cyan Fluorescent Protein (eCFP) and enhanced Yellow Fluorescent Protein (eYFP) that act as FRET partners [20]. Using site-directed mutagenesis, several FLIPPi affinity variants were generated, covering a wide range of physiological Pi concentrations with in vitro determined *K*_d_ (dissociation constant) values between 770 nm to 30 mM. The low-affinity FLIPPi-30m and high-affinity FLIPPi-5µ, with *K*_d_ values of 30 mM and 5 µM, respectively, were expressed in mammalian cells. Analysis in mammalian CHO cell line showed FRET (CFP/YFP) ratio change in response to Pi buffer perfusions in Pi-starved cells, revealing that FLIPPi sensors are suitable for real-time monitoring of Pi metabolism in living cells [20]. More recently, another generation of FLIPPi sensors, named cpFLIPPi, were obtained by substitution of eYFP with a circularly permuted (cp) form of the fluorescent protein Venus, reported to increase the magnitude of Pi-dependent FRET responses. In addition, mutagenesis of their sensor PiBP component generated new *K_d_* values ranging from 80 µM to 11 mM [21]. The cpFLIPPi-6.4m sensor, with *K_d_* value of 6.4 mM, was expressed and analyzed in the nematode *Caenorhabditis elegans* [22] and in the plant *Arabidopsis thaliana* (Arabidopsis) [21]. In *C. elegans*, the sensor was expressed in different cells and tissues, and upon injection of Pi buffer into intestinal cells, it showed in vivo FRET response to changes in Pi concentration, being also responsive to food deprivation [22]. In Arabidopsis, the sensor was constitutively expressed in a mutant deficient in transgene silencing to minimize potential loss of fluorescent signals caused by post-transcriptional gene silencing [15,21]. The FRET response allowed monitoring cytosolic Pi dynamics in root cells in response to Pi deprivation and resupply. In addition, a plastid-targeted form of the sensor was responsive to accumulation of Pi in plastids [21]. Here, we tested whether FLIPPi sensors can be expressed and analyzed in wild-type Arabidopsis plants, instead of having to use a mutant deficient in transgene silencing. In addition, we analyzed the FRET response to Pi-buffer perfusions in roots of the Arabidopsis lines expressing the low- and high-affinity FLIPPi sensors (FLIPPi-30m and FLIPPi-4µ, respectively), which so far were only tested in mammalian cell lines [20].

## 2. Results

### 2.1. In Vitro Analysis of Pi-Dependent FRET Response in Purified FLIPPi Sensors

**Figure 1:**
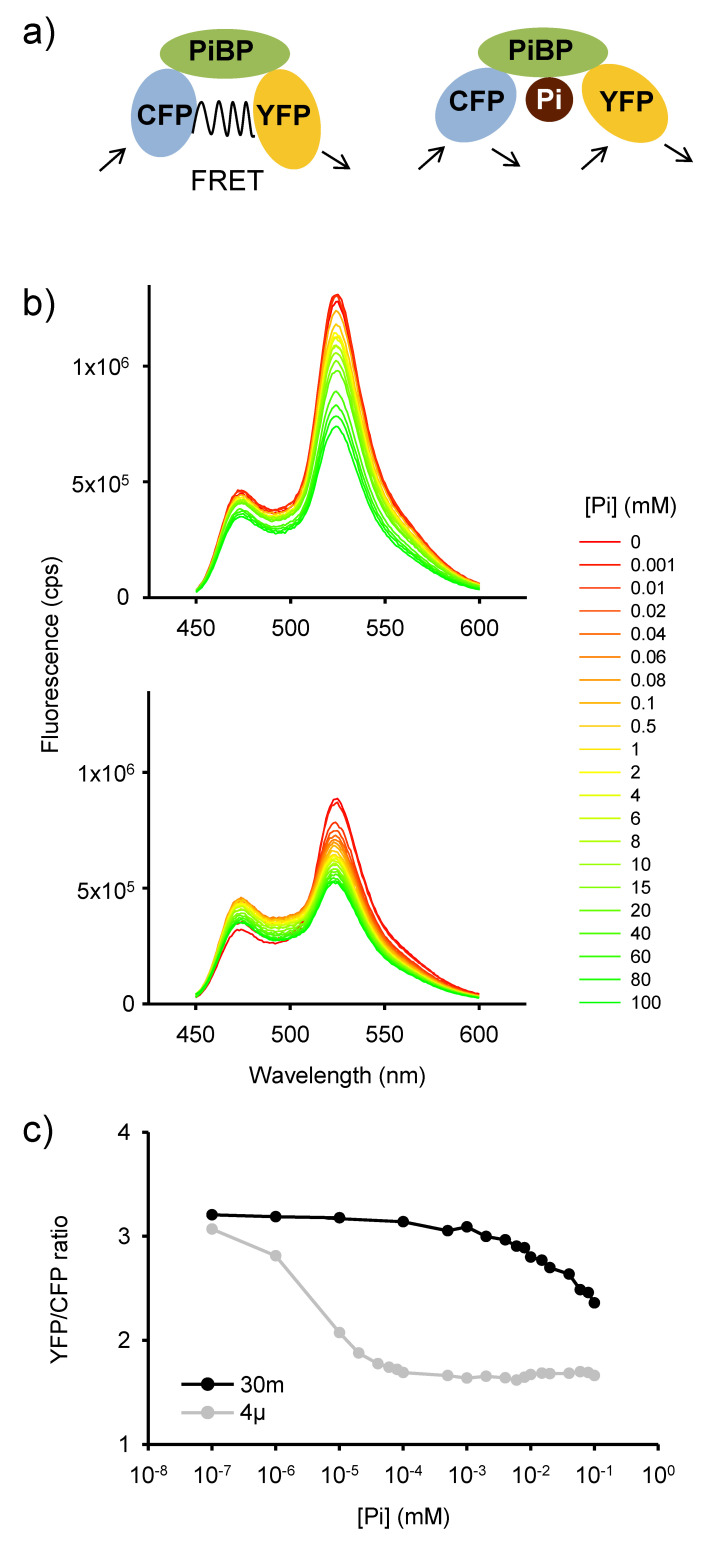
Pi-dependent changes in FRET ratios of purified FLIPPi Pi sensors. (**a**) Simplified scheme of FLIPPi FRET-based sensors [20] depicting the Pi binding bacterial periplasmic Binding Protein (PiBP) fused to Cyan Fluorescent Protein (CFP) and Yellow Fluorescent Protein (YFP) as FRET partners. (**b**) In vitro emission spectra of the low-affinity FLIPPi-30m and high affinity FLIPPi-4µ protein sensors in response to a series of Pi buffer solutions with Pi concentrations between 1.0 × 10^−3^ and 1.0 × 10^2^ mM; CFP and YFP emission peaks correspond to a wavelength of approximately 470 and 520 nm, respectively. Fluorescence intensity was measured in counts per second (cps). Measurements were performed with at least 3 sets of independently purified protein. (**c**) In vitro ligand-dependent FRET (YFP/CFP) ratio change in response to Pi buffer solutions, calculated from (b), for FLIPPi-30m (30m) and FLIPPi-4µ (4µ) sensor proteins.

To investigate whether the previously described FLIPPi sensors, low-affinity FLIPPi-30m and the high-affinity FLIPPi-4µ, with in vitro calculated *K_d_* values of 30 mM and 4 µM, respectively [20], can be expressed in Arabidopsis, we started by confirming that the purified FLIPPi-30 and FLIPPi-4µ sensor proteins yielded Pi-dependent FRET responses equivalent to those reported previously. The FLIPPi sensors contain a Pi binding PiBP, fused to eCFP and eYFP, that act as FRET partners, and upon Pi binding the YFP/CFP FRET ratio decreases [20] (Figure 1a). The purified FLIPPi-30m and FLIPPi-4µ sensor proteins yielded Pi-dependent FRET responses that showed a variation in the emission pattern, i.e., in the CFP and YFP peaks, with increasing Pi concentration (Figure 1b). The corresponding YFP/CFP FRET ratio change in relation to Pi concentration was calculated for FLIPPi-30m and FLIPPi-4μ proteins (Figure 1c), and the ligand-dependent FRET ratio change were in line with the previously established dynamic range, corresponding to between 10% and 90% saturation of the sensor, of 3–170 mM for FLIPPi-30m and 0.4-25 µM for FLIPPi-4μ, respectively [20].

### 2.2. Expression of FLIPPi Sensors in Wild-Type Arabidopsis

**Figure 2:**
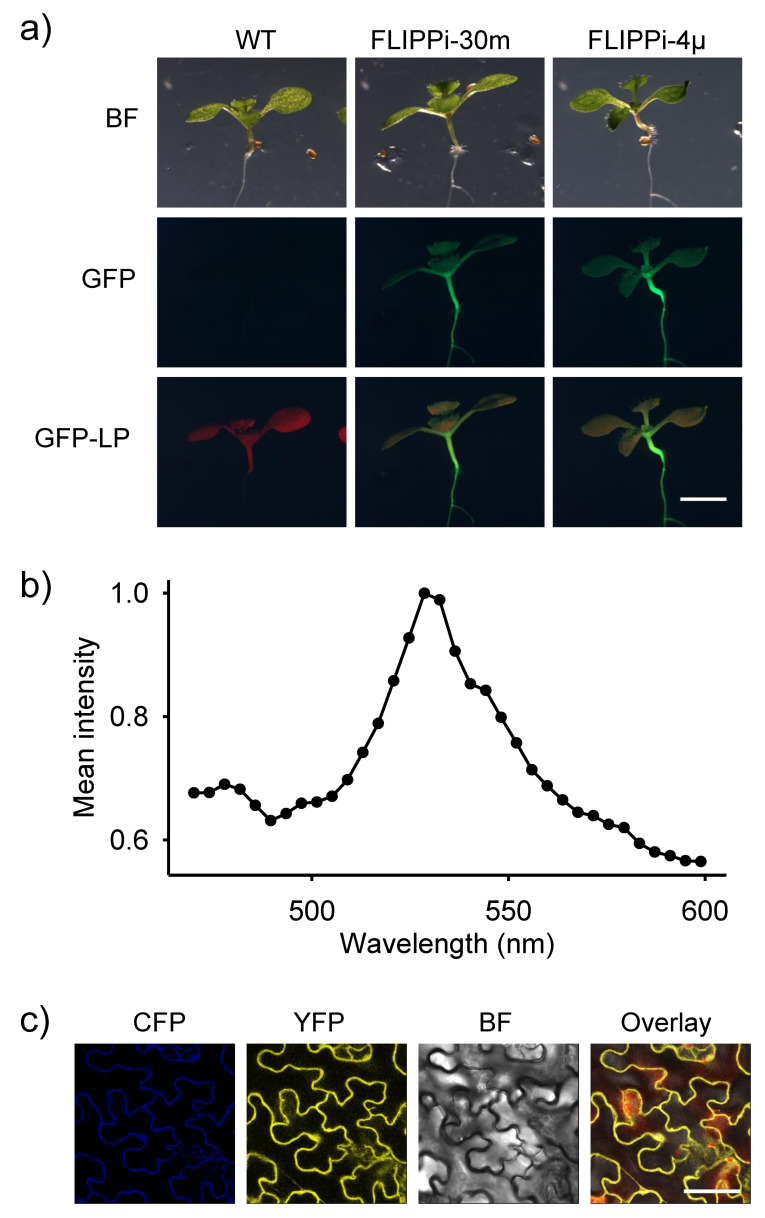
Expression of FLIPPi-30m and FLIPPi-4µ sensors in wild-type Arabidopsis. (**a**) Images from fluorescent microscopy of 12-day-old seedlings of Arabidopsis wild-type (WT), FLIPPi-30m and FLIPPi-4μ lines, observed with bright field (BF) and GFP filters (GFP, GFP-longpass). Scale bar: 2 mm (**b**) Emission spectrum (lambda-scan, λ) of leaf cells from 12-day-old seedlings of Arabidopsis FLIPPi-4µ line showing the CFP and YFP emission peaks, corresponding to a wavelength of approximately 470 and 520 nm, respectively. (**c**) Image from the CSLM-lambda (λ) scan in (b) showing the CFP, YFP, bright field (BP) and overlay channels. The same emission spectra (lambda-scan, λ) were obtained with seedlings of all independently transformed T3 generation Arabidopsis FLIPPi-4µ and FLIPPi-30m lines. Scale bar: 40 µm.

Next, we performed *Agrobacterium*-mediated stable transformation of wild-type Arabidopsis (Col-0) plants with the low-affinity FLIPPi-30m and high-affinity FLIPPi-4μ sensors, respectively, under control of the CaMV 35S promoter. Our results show that the attempt to express the FLIPPi sensors in wild-type Arabidopsis succeeded, with T1 generation transgenic lines selected to T3 generation homozygous lines. Plants and seedlings from the Arabidopsis FLIPPi lines were phenotypically indistinguishable from the Arabidopsis wild-type. The fluorescence of the FRET sensors was visualized at the fluorescence microscope (Figure 2a) and confirmed with the Confocal Laser Scanning Microscope (CLSM). A CLSM-lambda (λ) scan analysis showed the expected CFP and YFP fluorophore emission spectra, i.e., approximately 470 nm and 520 nm, respectively (Figure 2b,c). A CLSM-time (t) scan analysis, with the same settings, clearly showed cytoplasmic streaming indicating the presence of the genetically-encoded sensors in the plant cell cytosol (data not shown). These results confirm the constitutive expression of the low affinity FLIPPi-30m and high affinity FLIPPi-4µ sensors in the wild-type Arabidopsis.

### 2.3. Live Imaging of FRET Response to Pi Buffer Perfusions in Arabidopsis Roots

**Figure 3:**
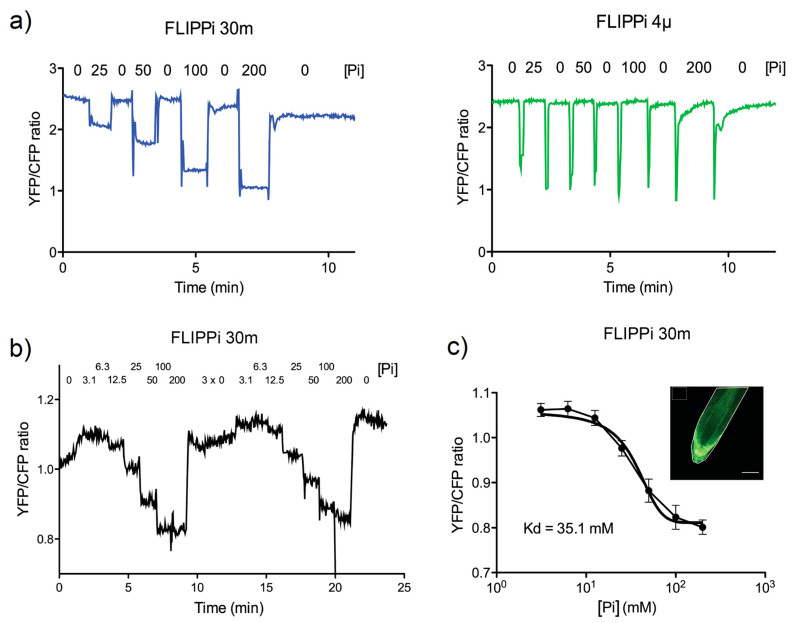
FRET response to Pi perfusions in roots of Arabidopsis FLIPPi sensor lines. (**a**) Time course of the effect of a series of Pi buffer perfusions with increasing Pi concentration (25; 50; 100; 200 mM), alternated with perfusion with Pi-free buffer, on the FRET (YFP/CFP) emission ratio changes in roots from 8–12-day-old seedlings of Arabidopsis FLIPPi-30m and FLIPPi-4μ lines. The analysis was performed with at least 3 independently transformed T3 homozygous FLIPPi-30m or FLIPPi-4µ lines, with ca. 3 seedlings analyzed per line. (**b**) Time course of the effect on FRET ratio changes of a series of Pi buffer perfusions with increasing Pi concentration (0; 3.1; 6.3; 12.5; 25; 50; 100; 200 mM) followed by perfusion with Pi-free buffer in roots of Arabidopsis FLIPPi-30m line. (**c**) Ligand-dependent FRET ratio change in response to Pi buffer solutions, calculated from (b). The curve and *K_d_* (mM) value were calculated with data points from each Pi concentration, corresponding to an average of ca. 10 YFP/CFP values. Top-right insert shows a CSLM overlay image of YFP/CFP channels with a seedling root used for perfusion analysis. The region of interest (ROI) measured in the analysis (covering the visible area of the root), and a ROI for background subtraction (square) are shown. Scale bar: 50 µm. The YFP/CFP emission ratio was calculated by dividing average pixel intensities of ROIs from each channel.

Finally, to assess whether it is possible to monitor in planta real-time variations of cellular Pi concentration with the Arabidopsis FLIPPi-30m and FLIPPi-4μ lines, we performed a live imaging analysis with Pi buffer perfusions in seedling roots. In short, roots were perfused with a series of Pi buffers with concentrations in the millimolar range using a set-up at the CLSM, which allows for solutions to be manually applied with a pipette on to the specimen and the excess liquid removed by continuous suction [19]. Roots of Arabidopsis FLIPPi-30m and FLIPPi-4μ lines were analyzed with a series of buffers with increasingly higher Pi concentration, alternated with a Pi-free perfusion. In the Arabidopsis FLIPPi-30m roots, the FRET (YFP/CFP) ratio decreased with increasing Pi concentration, and upon perfusion with Pi-free buffer, the FRET ratio had a fast and reversible response. On the other hand, the same perfusion analysis in the Arabidopsis FLIPPi-4μ roots showed a constant FRET ratio, irrespective of the Pi buffer concentration applied (Figure 3a). This suggests that at the millimolar Pi concentration range, the high-affinity FLIPPi-4μ sensor is likely to be saturated. Another perfusion analysis with a series of continuously increasing Pi concentration buffers was performed in the Arabidopsis FLIPPi-30m roots (Figure 3b). These data series were used to produce a curve of the ligand-dependent FRET ratio change in response to Pi buffer solutions for the FLIPPi-30m sensor and to determine the Pi binding affinity, which exhibited a *K_d_* value of 35 mM (Figure 3c).

## 3. Discussion

The FLIPPi family of Pi FRET-based sensors composed by the FLIPPi and cpFLIPPi sensors [20,21] comprises several affinity variants that cover a wide range of physiological Pi concentrations. Previously, the FLPPi sensor cpFLPPi-64m was expressed in Arabidopsis and its FRET response allowed live imaging of Pi in plants with cellular and subcellular resolution [21]. Past work expressed this FLIPPi sensor in Arabidopsis mutants deficient in transgene silencing, using the *rdr6* and *sgs3* mutants as background. The RNA-dependent polymerase RDR6 and the coiled-coil protein SGS3 are part of the pathway required for transgene-induced silencing [15]. This mutant background was used to minimize potential loss of fluorescent signals caused by post-transcriptional gene silencing [15,21]. Here, we instead attempted to stably transform the FLIPPi sensors in wild-type Arabidopsis. We obtained independent transgenic lines for each, the low-affinity FLIPPi-30m and high-affinity FLIPPi-4µ sensors, which exhibited strong fluorescence signal and were suitable for the analysis of FRET response to Pi buffer perfusions in T3 generation lines. Our results indicate that stable transformation in Arabidopsis with wild-type background can be an option for expressing and studying FLIPPi family sensors, which may be preferable to using Arabidopsis with a mutant background, to avoid possible undesired effects of the transgene silencing mutation. Supporting this conclusion, [17] could also analyses a glucose FRET-based sensor expressed in the T2 generation of wild-type Arabidopsis seedling roots. While we analyzed the T3 generation, we observed that the fluorescence signal intensity in Arabidopsis FLIPPi-4µ line became very weak in the T4 generation. By comparison, the FLIPPi-30m line maintained the same signal intensity, suggesting that post-transcriptional gene silencing likely affects the sensors differently.

These low-affinity FLIPPi-30m and high-affinity FLIPPi-4µ sensors were previously shown to be suitable for real-time monitoring of Pi metabolism and homeostasis in mammalian cell lines [20]. Here, we expanded this investigation to test whether these two FLIPPi sensors can be used for real-time monitoring of Pi concentration changes in plant cells as well. The plant cytosolic Pi concentration were estimated in the micromolar to low millimolar range, depending on the cell or tissue type, plant Pi-supply and methodology used [3,23,24,25]. The low-affinity FLIPPi-30m sensor, with a dynamic range of 3–170 mM, is suitable for tracking Pi changes in plant cells. The high affinity FLIPPi-4µ sensor, with a dynamic range of 0.4–25 µM, is expected to display substrate binding saturation in plant cells, as previously shown in mammalian cells [20], being used as an internal control. In order to monitor real-time Pi concentration changes in these Arabidopsis FLIPPi lines, we used a setup for live-cell microscopy with perfusion experiments at high spatial and temporal resolution, previously reported [19].

The live imaging analysis of seedling roots from the Arabidopsis FLIPPi-30m line showed FRET ratio changes in response to the Pi-buffer perfusions, with a clear Pi-dependent reversible FRET dynamic. The Pi buffer concentrations used, in the low millimolar range, overlap with the dynamic range of the FLIPPi-30m sensor. The analysis of seedling roots from the Arabidopsis FLIPPi-4µ line, on the other hand, showed no FRET response, in line with the expected substrate binding saturation upon Pi buffer perfusion, considering the low micromolar dynamic range of this high-affinity sensor [20]. Together, these results support that the live imaging analysis of FRET response in plant cells correspond to a Pi-specific response. Previously, the substrate specificity and pH sensitivity of the FLIPPi sensors were tested and, within the physiological range, appeared to be non-responsive to nitrate and sulphate, and also unaffected by pH changes in the 6.8 and 7.6 range [20]. Our Pi-buffer perfusion analysis of the Arabidopsis FLIPPi-30m seedling roots further allowed the in planta calculation of the Pi binding affinity, which exhibited a similar *K_d_* value to the one earlier calculated in vitro (*ca.* 35 and 30 mM, respectively, [20]). This indicates that the performance of the FLIPPi-30m protein as a Pi sensor is comparable between the in vitro analysis with the purified protein and the analysis in plant cells with the genetically-encoded and constitutively expressed protein. A potential problem in our in vivo analysis is that the specimen preparation for the live-cell microscopy applied medical adhesive to immobilize the seedling roots, and the existence of cells with compromised integrity cannot be ruled out [19]. Nonetheless, such an in vivo approach is still useful to test if the binding affinity and substrate specificity of a genetically encoded nanosenor in a cellular environment are similar to the ones calculated in vitro. In that perspective, our results showing similar Pi binding affinity between analysis in root cells and with purified protein in vitro is a relevant finding for the future use of FLIPPi family sensors.

## 4. Conclusions

Live imaging of Pi levels in plant cells using the FLIPPi family of Pi FRET-based sensors [20,21] can be a valuable tool to study Pi transport and homeostasis mechanisms in plants. Our analyses of two FLIPPi sensors, which were not previously tested in plants, indicated that stable transformation in Arabidopsis with wild-type background can be an option for expressing FLIPPi family sensors. In particular, we showed FRET response in root cells expressing the FLIPPi-30m low-affinity sensor, with a similar Pi binding affinity to that in in vitro analysis. Our results reinforce the potential of the FLIPPi family sensors to study the molecular basis of Pi homeostasis in plants. The possibility for live imaging of Pi levels in cells could, for example, be a complementary tool to support the analysis of genes with a putative function on Pi transport and homeostasis, and to assist the selection of lines with improved Pi uptake or Pi-use efficiency traits. In the future, FLIPPi family sensors could also be used in crop research and lead towards solutions for a more sustainable use of Pi fertilization.

## 5. Materials and Methods

### 5.1. Plasmid Construction and Plant Transformation

The Pi sensor affinity variants FLIPPi-30m and FLIPPi-4μ were previously described [20]. To generate overexpression constructs for plant transformation, FLIPPi-30m and FLIPPi-4µ sequences were amplified from pRSET-FLIPPi-30m and pRSET-FLIPPi-4µ constructs [20], respectively, and cloned into the donor vector pENTR Directional TOPO (Invitrogen, Waltham, MA, USA). The fragments were amplified using Phusion polymerase (Finnzymes, Espoo, Finland), with PCR conditions as recommended by the manufacturer, and using a forward primer with 5′-CACC overhang as recommended for directional TOPO Cloning (Invitrogen, Waltham, MA, USA). The primers used were 5′-CACCATGGTGAGCAAGGGCGAG-3′ and 5′-TTACTTGTACAGCTCGTCCATGCCG-3′. The obtained entry clones were further cloned into a destination vector by in vitro site-directed recombination into the binary vector pEarlyGate-100 [26], placing genes under control of the constitutive cauliflower mosaic virus (CaMV) 35S promoter. The expression clones pEarlyGate-FLIPPi-30m and pEarlyGate-FLIPPi-4µ were verified by digestion analysis and sequencing, transformed by electroporation into *Agrobacterium tumefaciens* strain C58C1 and subsequently transformed into Arabidopsis wild-type plants (Columbia, Col-0) by floral dipping [27]. Transgenic plants were selected by BASTA resistance and 5 independent lines per each FLIPPi sensor variant construct were selected to homozygous T3 generation. At least 3 independently transformed homozygous lines per construct were analyzed. They were referred to as FLIPPi-30m and FLIPPi-4µ lines. The expression of the FLIPPi sensor was verified by observation of the emission spectra (lambda-scan, λ) in seedlings of all independently transformed T3 generation Arabidopsis FLIPPi-4µ and FLIPPi-30m lines.

### 5.2. In Vitro Analysis of FLIPPi Proteins

The pRSET-FLIPPi-30m and pRSET-FLIPPi-4µ constructs were transformed into *E. coli* BL21-Gold (DE3) (Strategene, San Diego, California, USA) by electroporation. Protein expression and purification was performed as previously described [20]. The emission spectra of FLIPPi-30m and FLIPPi-4µ proteins were obtained using a spectrofluorometer (FluoroMax-4, HORIBA Scientific, Kyoto, Japan) with an excitation wavelength of 425 nm, and with emission recorded from 450 to 600 nm (every 1 nm). Ligand titration curves were obtained using a microplate reader (Fluostar, BMG Labtech, Offenburg, Germany) with an excitation filter of 410/12 nm, and emission filters of 470/12 nm and 520 nm, for CFP and YFP, respectively. All assays were performed with a series of Pi buffer solutions with Pi concentrations between 1.0E-06 and 1.0E-01 M, with 20 mM Tris-HCl and pH 7.0, as described by [20]. Measurements were performed with at least 3 sets of independently purified protein.

### 5.3. Plant Growth Conditions

Seeds from the Arabidopsis FLIPPi-30m and FLIPPi-4µ lines, were surface-sterilized using vapor-phase seed sterilization and sown on plates with ½ MS media (Duchefa Biochemie, Haarlem, The Netherlands) supplemented with 0.5% sucrose and adjusted to pH 5.8. Prior to germination, seeds were stratified for 3 days in a cold room at 4°C in the dark to promote uniform germination. MS plates were placed in a growth chamber with 16/8 h light/dark cycle, with 125 µmol m^−2^ s^−1^ white light, 22/20 °C light/dark temperature, and 70% relative humidity. Eight- to 12-day-old seedlings were used for FRET analysis with Pi buffer perfusions in roots.

### 5.4. Live Imaging and FRET Analysis with Pi Buffer Perfusions

Imaging analysis was performed with confocal laser scanning microscopy (CLSM) using a Leica TCS SP2 and SP5-X microscopes (Leica Microsystems), with an HCX PL APO 20x /0.70 water/immersion objective or an HCX PL APO 40x /0.70 water/immersion objective for root or leaf observations, respectively. The Argon laser line was 458 nm and the emission settings were recorder for CFP (470–510 nm), YFP (520–560 nm), far red (chloroplast auto-fluorescence) and transmission (bright field). For lambda-series recording (xyλ), the emission was recorded from 470–600 nm. For Pi buffer perfusion experiments, a time series recording (xyt) was used. Specimen preparation prior to Pi-buffer perfusion was done by immobilizing the root on a microscope slide, using medical adhesive (Hollister no. 7730, USA), without detachment from the seedling. The measurements were performed with at least 3 independently transformed T3 homozygous FLIPPi-30m or FLIPPi-4µ lines, with ca. 3 seedlings analyzed per line. The analysis was performed with a series of Pi buffer solutions with concentrations between 0 and 200 mM (0; 3.1; 6.3; 12.5; 25; 50; 100; 200 mM), with either 20 mM Tris-HCl or 20 mM MOPS and pH 7.0. A suction device, connected to a peristaltic pump, was placed with the tip almost touching the dipping drop of the sample. While adding the perfusion solution with a pipette tip from the opposite side of the slide, excess solution was removed by suction, resulting in replacement of the solution, as described by [19]. The specimen was perfused with 1 mL of Pi buffer of increasing Pi concentration, followed by 1 mL of Pi-free buffer, applied with approximately 2.5 min interval between two applications. The recordings were taken in a region of interest (ROI) covering the visible seedling root, ranging from the root tip to the elongation zone, ca. 1000 µm from root tip. An ROI for background subtraction was used. The YFP/CFP emission ratio was calculated by dividing the average pixel intensities of ROIs from each recorded YFP and CFP channels, respectively.

### 5.5. Data Analysis

Selection of perfusion experiments for data analysis was done when the optical sections did not show focus shift or drifting during the recording, and had a relatively stable initial ratio baseline before any changes of the perfusion buffer. Imaging data were analyzed using the open source software ImageJ with the LOCI bio-formats plugin: http://rsb.info.nih.gov/ij/index.html. The YFP/CFP emission ratio from live imaging analysis was calculated by dividing average pixel intensities of regions of interest (ROIs) from each channel, and data was presented using Prism from GraphPad Software as described by [19]. The ligand-dependent FRET ratio change curves in response to Pi and the *K_d_* (mM) value were calculated with data points from each Pi concentration, corresponding to an average of ca. 10 YFP/CFP values, by a non-linear fit (variable slop) to an inhibitor dose response equation, using Prism from GraphPad Software.
